# Assessment of the knowledge, attitudes, and risk perceptions of pharmacy students regarding HIV/AIDS

**DOI:** 10.3389/fpubh.2024.1499898

**Published:** 2025-01-08

**Authors:** Wael Mansy, Wajid Syed, Salmeen Babelghaith, Mohamed N. Al-Arifi

**Affiliations:** Department of Clinical Pharmacy, College of Pharmacy, King Saud University, Riyadh, Saudi Arabia

**Keywords:** acquired immune deficiency syndrome, human immunodeficiency virus, knowledge, attitudes, perceptions

## Abstract

**Background:**

We recognize AIDS and HIV as serious public health concerns. One of the primary roles of pharmacists is to counsel patients, which is critical in improving patient care outcomes. Therefore, having an adequate understanding of HIV among undergraduates helps them at their practice sites. This study aimed to assess the knowledge, attitudes, and risk perceptions of pharmacy students regarding HIV/AIDS.

**Methods and materials:**

We conducted a cross-sectional study among pharmacy students from December 2023 to April 2024 using structured questionnaires. We collected the data from randomly selected students currently enrolled in the College of Pharmacy at King Saud University in Riyadh, Saudi Arabia. The questionnaire consisted of four parts focusing on students’ demographics, knowledge, attitude, and risk perception toward HIV/AIDS. The data was analyzed using SPSS software, with Chi-squared and Fisher’s tests utilized to determine associations between categorical variables at a significant level of *p* < 0.05.

**Results:**

A total of 395 pharmacy students completed an online survey. The total mean knowledge score was 6.1 ± 2.8 (Range: 0–13; Median = 6). The majority of students had low knowledge of HIV/AIDS (79.0%) and, a neutral attitude toward HIV/AIDS (61.5%) and only 13.2% of students showed a positive attitude, and 25.3% had a negative attitude toward HIV/AIDS. In addition, 35% of students thought that patients with HIV should be quarantined, 45% of students were concerned about workplace transmission of HIV and their safety on the job, and 41% of them would prefer not to take care of patients with HIV. Furthermore, students aged between 23 and 26 years had a high knowledge level compared to other groups (*p* < 0.001). Moreover, intern students showed a high level of knowledge of HIV/AIDS compared to other students (*p* < 0.001). The majority of pharmacy students had a neutral perception of the risk of HIV/AIDS (63.0%). Only 6.1% of pharmacy students reported a positive perception of the risk of HIV/AIDS.

**Conclusion:**

In conclusion, there was a dearth of knowledge of HIV/AIDS and neutral attitudes and perceptions of the disease (HIV) among pharmacy students. When compared to other age groups, students between the ages of 23 and 26 had a high degree of knowledge. These findings suggest that specific strategies, such as integrating education on this topic into the pharmacy curriculum and running awareness campaigns for undergraduate students, are necessary.

## Introduction

Around the world, Acquired Immune Deficiency Syndrome (AIDS) and the human immunodeficiency virus (HIV) are acknowledged as serious public health concerns (1). By the end of 2023, an estimated 39.9 million people were HIV positive, and 65% of them belonged to the WHO African region (1–3). World Health Organization recently reported that an estimated 630,000 people died from HIV-related causes and an estimated 1.3 million people acquired HIV (2). The Middle East and North Africa (MENA) region has a low number of HIV infections as compared to other regions, but recent studies have shown that HIV incidence is rising, especially among high-risk groups (1). The prevalence of HIV has been lower in many Middle Eastern countries than in other regions because of social, cultural, and religious taboos (4).

The Kingdom of Saudi Arabia, which occupies the majority of the Arabian Peninsula, is one of the largest nations in the Arab world. With 61.2% of the world’s male population, it is the biggest country in the Middle East and Asia (5). In total, 1.72 million students were enrolled in various education programs out of the 3.5 million individuals in the 18–24 age group (6). Higher risk sexual activities, or those that greatly raise the risk of STIs and HIV, are most likely to occur during these ages. Additionally, the literature has revealed that sex before marriage among students has been on the rise globally (7). In Saudi Arabia, the first incidence of HIV was reported in early 1984. By 2013, around 1,509 patients had been diagnosed with HIV infection (8). However, in 2022, the prevalence of HIV was very low, estimated at 0.1% (9).

One of the main pillars in the battle against HIV/AIDS is understanding individuals’ knowledge and level of awareness about the illness (10). The most common problem for people living with HIV/AIDS is the public’s ignorance or misinformation about the disease (10). This, in turn, contributes to discrimination against them, leading to issues with disclosure, social isolation, access to antiretroviral therapy, and psychological support (10). Therefore, it is essential to understand HIV transmission, and having adequate knowledge may help individuals avoid the possibility of infection. Earlier literature has shown limited knowledge of HIV among non-medical students (11). For example, a study conducted by Terra et al. (11) in Africa among undergraduate students revealed an overall HIV knowledge score of 14, with medical students having significantly higher knowledge than non-medical students. In Saudi Arabia, a previous study among medical students showed a moderate level of HIV knowledge and negative attitudes toward HIV (12). Another recent study among pharmacy students found lower levels of knowledge about HIV/AIDS, with half of the students not realizing that antivirals cannot treat HIV/AIDS and feeling they did not receive enough education on HIV (3). Previous studies also found that the majority of medical students were unaware of HIV transmission and prevention (12). On the other hand, a study conducted in Canada and Qatar found that while students are typically aware and have positive views, Qatari students had severe misconceptions, and attitudes and beliefs differed significantly between students in Canada and Qatar (13). Additionally, earlier literature revealed inadequate aspects of HIV knowledge among undergraduate students (11). Therefore, this study aimed to assess the Knowledge, Attitudes, and Risk Perceptions of Pharmacy Students Regarding HIV/AIDS.

## Methods

We conducted a cross-sectional study among pharmacy students between December 2023 and April 2024 using structured questionnaires. We collected the data from randomly selected students currently pursuing their courses at the College of Pharmacy in a Saudi university in Riyadh, Saudi Arabia. The simple random sampling strategy involves randomly selecting individuals from a wider population. Every member of the population has an equal chance of selection. The study encompassed Saudi nationals completing a college-level equivalent pharmacy degree, aged 18 years and above, who can provide informed consent and regularly attend the university. We excluded non-pharmacy students who did not meet the inclusion criteria. Furthermore, before data collection, the study questionnaires were reviewed and approved by the research committee College of medicine at King Saud university.

A pre-validated self-administered anonymous survey was developed from a pervious study aimed to assess the knowledge, attitude, and perception of pharmacy students (14). The questionnaire consisted of four parts that were completed in English format and were intended to collect data on student’s demographics. Knowledge, attitude, and risk perception toward HIV/AIDS (14). The first part included gender, age, and year of study. The second component assessed the students’ knowledge of HIV/AIDS with 15 questions that may be answered “yes,” “no,” or “I do not know.” The third part assessed the student’s general attitude toward HIV/AIDS and had five items in the following formats: “strongly agree,” “agree,” “neutral,” “disagree,” and “strongly disagree (14). The fourth part assessed the student’s risk perception toward HIV/AIDS and had five statements presented in “strongly agree,” “agree,” “neutral,” “disagree,” and “strongly disagree.

After the initial draft of the questionnaire, it was subjected to a panel of experts (two professor form clinical pharmacy apartment and a researcher) for their feedback about the suitability of the questionnaires, later a pilot study was carried out to check the content, flow, and time taken to answer. The pilot study was conducted among randomly selected students (30). The findings of the pilot study were not included in the main analysis. The reliability was determined using Cronbach’s alpha, which was found to be 0.78 for the knowledge items, while for the attitudes it was 0.72, and risk perceptions 0.69, suggesting that questionnaires are valid and reliable to carry out the study.

However, a knowledge score was calculated by giving a score of 1 For every correct answer (“yes”), while a score of zero is given for wrong answer (“no” and “I do not know”). Next, utilizing Bloom’s cut-off point, the student’s total knowledge score was categorized, with a score of 12–15 reflected high a score of 9–11.9 was moderate, and less than 9 was low (15). The attitude and risk perception questions were composed of 5 items, with a total score of 25. For the positive items, the score for the Likert scale was 5 (strongly agree) to 1 (strongly disagree), and for the negative questions the score was the reverse of the positive items. Based on the cut-off point (15), the students’ total attitude and perception scores were categorized as follows: Bloom’s cutoff categories for the attitudes, and risk perception scores.

**Table:** 

Items	Category	Scores
Attitude	Positive	20–25
	Neutral	15–19
	Negative	<15
Risk perception	Positive	20–25
	Neutral	15–19
	Negative	<15

### Data analysis

The data was analyzed using Statistical Package for Social Sciences version 26.0 (SPSS Inc., Chicago, IL, United States). Chi-squared and Fisher’s test were used to find the association between categorical variables at *p* < 0.05 significant level.

## Results

We approached 500 students out of the 395 who completed, resulting in a response rate of 79% (*n* = 500). Most of the students were male (55.9% and 44.1%) were female. The majority of students were in the second year of their Pharm D degree course (22.0%), followed by the third year (18.2) and first year (14.2%) as shown in Table 1.

**Table 1:** Sociodemographic data of the students (*n* = 395).

Variables	*N* (%)
Gender	
Male	221 (55.9)
Female	174 (44.1)
Age	
18–22	139 (35.2)
23–26	206 (52.2)
27–30	35 (8.9)
>30	7 (1.8)
Missing	8 (2.0)
Year of study	
1st year	56 (14.2)
2nd year	87 (22.0)
3rd year	72 (18.2)
4th year	56 (14.2)
5th year	32 (8.1)
Intern	45 (11.4)
Postgraduate	47 (11.9)

### Pharmacy students’ knowledge of HIV/AIDS

This study evaluated the HIV/AIDS knowledge of pharmacy students, as depicted in Table 2. The average knowledge score was 6.1 ± 2.8. The correct answer rate on the HIV/AIDS knowledge questionnaire was 41.0% (6.1/15*100). Furthermore, the majority of pharmacy students demonstrated low knowledge of HIV/AIDS (79.0%), indicating insufficient awareness. Most pharmacy students (71.1%) correctly identified that HIV/AIDS can be managed with antiretroviral therapy. Approximately two-thirds of pharmacy students (59.7%) recognized the main transmission routes of HIV/AIDS. Additionally, 52.9% of pharmacy students acknowledged that HIV/AIDS can be passed from an infected mother to her child. Around half of pharmacy students were aware that individuals with HIV/AIDS are ineligible to donate blood. Approximately 34% of pharmacy students knew that antiviral medications like acyclovir, ribavirin, and amantadine are not effective in treating HIV/AIDS. Only 27.3% of pharmacy students disagreed with the notion that HIV infection can progress to AIDS within a year.

**Table 2:** Pharmacy students’ knowledge about HIV/AIDS.

Variables	Yes *n* (%)	No *n* (%)	I do not know *n* (%)	Correct answer *n* (%)
Can HIV/AIDS be transmitted from infected mother to child?	209 (52.9)*	117 (29.6)	69 (17.5)	209 (52.9)
Can HIV be transmitted via air and contact with water?	94 (23.8)	222 (56.2)*	3 (79)	222 (56.2)
Can HIV be transferred through social contacts like sharing clothes? Cups/plates/spoon/glass, bathrooms, shaking hands and kissing?	123 (31.1)	193 (48.9)*	79 (20.0)	193 (48.9)
Major routes of transmission are unprotected sex, unscreened blood, occupational exposures, and intravenous drug use	236 (59.7)	78 (19.7)	81 (20.5)	236 (59.7)
Do you think HIV can be completely cured with available antiretroviral therapy?	114 (28.9)	281 (71.1)		281 (71.1)
Non-nucleosides/nucleosides reverse transcriptase inhibitors and Protease inhibitors are the most widely available classes of HIV medications	203 (51.4)	79 (20)	113 (28.6)	203 (51.4)
Antiviral drugs like Acyclovir, Ribavirine, and Amantadine can be used to treat HIV/AIDS.	152 (38.5)	133 (33.7)	110 (27.8)	133 (33.7)
Can HIV/AIDS patients donate blood?	96 (24.3)	199 (50.4)	100 (25.3)	199 (50.4)
Is post-exposure prophylaxis using antiretroviral recommended?	177 (44.8)	90 (22.8)	128 (32.4)	177 (44.8)
Is HIV and AIDS are similar things?	149 (37.7)	147 (37.2)	99 (25.1)	147 (37.2)
Does avoiding mosquito bites prevent HIV?	114 (28.9)	178 (45.1)	103 (26.1)	178 (45.1)
Osteomyelitis, typhoid, and Endocarditis are the major infections in HIV patients causing deaths.	193 (48.9)	80 (20.3)	122 (30.9)	80 (20.3)
Can food sharing with HIV-infected individuals transmit HIV?	131 (33.2)	183 (46.3)	81 (20.5)	183 (46.3)
Can HIV infection develop into AIDS within a year?	135 (34.2)	108 (27.3)	152 (38.5)	108 (27.3)
Contact with feces, urine, and saliva can cause HIV.	154 (39.0)	143 (36.2)	98 (24.8)	143 (36.2)

Table 3 shows the Correlation of knowledge level with the socio-demographic characteristics of the participants. There were no significant differences between gender and knowledge levels. There was a significant difference between age groups and knowledge levels of HIV/AIDS. Where students aged between 23 and 26 years had a high knowledge level compared to other groups (*p* < 0.001). Moreover, intern students showed a high level of knowledge of HIV/AIDS compared to other students (*p* < 0.001).

**Table 3:** Correlation of knowledge level with socio-demographic characteristics of the participants.

Variables	Knowledge levels			*p*-value
	High (*n* = 17) *n* (%)	Moderate (*n* = 66) *n* (%)	Low (*n* = 312) *n* (%)	
Gender				0.728
Male	8 (47.1)	36 (54.5)	177 (56.7)	
Female	9 (52.9)	30 (45.5)	135 (43.3)	
Age				<0.001*
18–22	0	18 (27.7)	121 (39.3)	
23–26	11 (78.6)	31 (47.7)	164 (53.2)	
27–30	2 (14.3)	11 (16.9)	22 (7.1)	
>30	1 (7.1)	5 (7.7)	1 (0.3)	
Missing	3	1	4	
Year of study				
1st year	0	3 (4.5)	53 (17.0)	<0.001*
2nd year	0	6 (9.1)	81 (26.0)	
3rd year	2 (11.8)	7 (10.6)	63 (20.2)	
4th year	0	9 (13.6)	47 (15.1)	
5th year	2 (11.8)	9 (13.6)	21 (6.7)	
Intern	8 (17.8)	15 (22.7)	22 (7.1)	
Postgraduate	5 (29.4)	17 (25.8)	25 (8.0)	
Total	17 (4.3)	66 (16.7)	312 (79.0)	

*Fisher-exact test.

### Attitude of pharmacy students about HIV/AIDS

This study reported that the most of students had a neutral attitude toward HIV/AIDS (61.5%). Only 13.2% of participants showed a positive attitude, and 25.3% had a negative attitude toward HIV/AIDS as presented in Table 4. Slight more than half of the participants agreed that they were qualified to treat and care for patients with AIDS. About 41% of pharmacy students said that they would prefer not to treat HIV patients. About half of pharmacy students expressed a readiness to help or care for HIV/AIDS patients in operating rooms and hospital wards. Only 47.1% of pharmacy students showed confidence that their professional education had adequately provided them with the basic information and skills needed to properly collaborate with HIV/AIDS patients (Table 5). Table 4 displays the association between attitude levels and gender, age groups, and year of study. Only there was a significant association between the year of study and attitude levels, the students studied in the second year showed a positive attitude than others (*p* = 0.043).

**Table 4:** Correlation of attitude level with socio-demographic characteristics of the participants.

Variables	Positive *n* (%)	Neutral *n* (%)	Negative *n* (%)	*P*-value
Gender				0.097
Male	23 (44.2)	145 (59.7)	53 (53.0)	
Female	29 (55.8)	98 (40.3)	47 (47.0)	
Age				0.150*
18–22	24 (47.1)	81 (33.5)	34 (36.2)	
23–26	26 (51.0)	135 (55.8) 22 (9.1)	45 (47.9)	
27–30	1 (2.0)	4 (1.7)	12 (12.8)	
>30			3 (3.2)	
Year of study				
1st year	7 (13.5)	33 (13.6)	16 (16.0)	0.043*
2nd year	13 (25.0)	54 (22.2)	20 (20.0)	
3rd year	12 (23.1)	45 (18.5)	15 (15.0)	
4th year	11 (21.2)	35 (14.4)	10 (10.0)	
5th year	1 (1.9)	25 (10.3)	6 (6.0)	
Intern	5 (9.6)	29 (11.9)	11 (11.0)	
Postgraduate	3 (5.8)	22 (9.1)	22 (22.0)	
Total	52 (13.2)	243 (61.5)	100 (25.3)	

*Fisher-exact test.

**Table 5:** Pharmacy student’s attitudes about HIV/AIDS.

Items	Strongly agree *n* (%)	Agree *n* (%)	Neutral *n* (%)	Disagree *n* (%)	Strongly disagree *n* (%)
Do you feel you are competent enough to provide treatment, care, and counseling for HIV/AIDS patients?	117 (29.6)	90 (22.8)	93 (23.5)	69 (17.5)	26 (6.6)
I would prefer not to take care of HIV patients.	62 (15.7)	101 (25.6)	106 (26.8)	80 (20.3)	46 (11.6)
I am willing to assist/take care of HIV patients in wards and operation theaters	80 (20.3)	115 (29.1)	101 (25.6)	57 (14.4)	42 (10.6)
Patients with HIV/AIDS should be nursed separately.	78 (19.7)	83 (21.0)	113 (28.6)	74 (18.7)	47 (11.9)
My professional education has provided me with enough education/information to work safely with AIDS patients.	98 (24.8)	88 (22.3)	97 (24.6)	71 (18.0)	41 (10.4)

Students’ year of study was significantly associated with attitudes toward HIV as shown in Table 4.

### Perceptions of pharmacy students on HIV/AIDS risk

The majority of pharmacy students had a neutral perception of the risk of HIV/AIDS (63.0%). Meanwhile, 6.1% of pharmacy students reported a positive perception of the risk of HIV/AIDS. About 42% of pharmacy students agreed that children with HIV should be educated in separate schools. Meanwhile, 33.5% of pharmacy students agreed that Patients with HIV should be quarantined. Roughly 45% of pharmacy students stated they were worried about getting HIV at work. Over half of pharmacy students (52.4%) often suggested that HIV testing should be voluntary for all healthcare staff and 57.2% of them suggested compulsory HIV testing for patients who were hospitalized for surgery (Table 6).

**Table 6:** Pharmacy students’ risk perceptions about HIV/AIDS.

Items	Strongly agree	Agree *n* (%)	Neutral *n* (%)	Disagree *n* (%)	Strongly disagree *n* (%)
HIV-infected children should be educated in separate schools	80 (20.3)	85 (21.5)	94 (23.8)	74 (18.7)	62 (15.7)
Patients with HIV should be quarantined	52 (13.2)	80 (20.3)	81 (20.5)	88 (22.3)	94 (23.8)
I worry about acquiring HIV at my workplace in my professional life	83 (21.0)	96 (24.3)	103 (26.1)	75 (19.0)	38 (9.6)
Initial HIV tests should be conducted for all patients admitted for surgical procedures.	110 (27.8)	97 (24.6)	98 (24.80)	56 (14.2)	34 (8.6)
In my opinion, all healthcare students and professionals should go for mandatory HIV testing.	134 (33.9)	92 (23.3)	76 (19.2)	62 (15.7)	31 (7.8)

A Fisher-exact test showed that there was only a significant correlation between risk perception items and year of study groups, however, the second-year students had a more positive perception of the risk of HIAIDS than other students (*p* = 0.001) as shown in Table 7.

**Table 7:** Correlation of perception level with socio-demographic characteristics of the participants.

Variables	Positive (*n* = 24) *n* (%)	Neutral (*n* = 249) *n* (%)	Negative (*n* = 122) *n* (%)	*P*-value
Gender				0.089
Male	18 (75.0)	141 (56.6)	62 (15.7)	
Female	6 (25.0)	108 (43.4)	60 (49.2)	
Age				0.585*
18–22	11 (45.8)	93 (37.7)	35 (30.2)	
23–26	12 (50.0)	130 (52.6)	64 (55.2)	
27–30	1 (4.2)	20 (8.1)	14 (12.1)	
>30		4 (1.6)	3 (2.6)	
Missing				
Year of study				0.001*
1st year	3 (12.5)	44 (17.7)	9 (7.4)	
2nd year	10 (41.7)	55 (22.1)	22 (18.0)	
3rd year	2 (8.3)	42 (16.9)	28 (23.0)	
4th year	1 (4.2)	41 (16.5)	14 (11.5)	
5th year	1 (4.2)	16 (6.4)	15 (12.3)	
Intern	6 (25.0)	27 (10.8)	12 (9.8)	
Postgraduate	1 (4.2)	24 (9.6)	22 (18.0)	
Total *n*(%)	24 (6.1)	249 (63.0)	122 (30.9)	

*Fisher-exact test.

## Discussion

Despite the numerous efforts of healthcare organizations and professionals, Acquired Immunodeficiency Syndrome (AIDS) remains one of the most prevalent health concerns worldwide and is driven by a variety of social, economic, and cultural factors. In this study, the mean knowledge score was low, with only one-fourth of the students correctly answering the knowledge questionnaire. These findings were comparable to earlier studies published in other countries (3, 11, 16). For example, similar data among medical students in Saudi Arabia revealed a mean HIV knowledge score of 11.62 and a mean HIV attitude score of 37.82. The findings concluded that there is a modest level of HIV knowledge among medical students and negative attitudes toward People Living with HIV (PLHIV) (12).

In Saudi Arabia, a previous study among pharmacy students showed similar outcomes and concluded that pharmacy students had lower knowledge about HIV/AIDS (the mean knowledge score was 8.22 out of 15) (3). Similarly in Africa, an earlier study reported that the overall knowledge score of students was 21% had a poor level of knowledge, (126)37% had average knowledge, and (145)42% had adequate knowledge (11). Among dental students, a previous study in Saudi Arabia revealed that 39.5% of the students showed low knowledge of safety regarding HIV (16). However, these findings were lower than earlier findings by Zhang et al. (17) among Chinese university students who reported higher levels of knowledge of 80.8%. Similarly, another study by Ma et al. (18) revealed very high levels of HIV cognitive knowledge among 91% of the students. The results of this study indicate that there may be variations in students’ knowledge and attitudes, which could be attributed to factors such as country and disease prevalence. While it is true that individuals in countries with higher disease prevalence tend to have higher levels of knowledge, the fact that HIV prevalence is very low in Saudi Arabia may contribute to the country’s low level of knowledge about the disease. Nonetheless, these results imply that educational authorities ought to move to advance the use of illness knowledge.

In this study, 33.7% of the students correctly answered that Antiviral drugs like Acyclovir, Ribavirine, and Amantadine can be used to treat HIV/AIDS. While previous study among pharmacy students reported that 60.8% of them did not realize that antivirals could not treat HIV/AIDS (3). The findings suggest that students need additional education and awareness of the diseases therefore educational institutions and health care planners should perform extra curriculum activities about these diseases to enhance HIV understanding. In this study knowledge of HIV was not significantly associated with the gender of the student, however, there was a significant difference between age groups and knowledge of HIV/AIDS. For example, students aged between 23 and 26 years had higher levels of HIV knowledge, compared to other age group students. In addition, intern students showed a higher level of knowledge of HIV/AIDS compared to other students (*p* < 0.001). In a previous study students’ HIV knowledge was correlated to student gender, nationality, marital status, and their grade (*p* < 0.01). In addition, female students had insufficient HIV knowledge (17).

Additionally, in the earlier study, 46.2% of students felt they received insufficient training on how to work safely and responsibly with people living with HIV/AIDS, and 20% expressed a reluctance to assist those living with the virus (3). Furthermore, nearly half of the students (45%) were concerned about contracting HIV in the health workplace, indicating a pressing need for training and reassurance about the reduced risk of workplace transmission, particularly among practicing pharmacists and other healthcare professionals. It is important to note that there is no risk in having usual social or professional interaction with an HIV-positive person. Droplets, urine, or saliva cannot spread HIV. However, to prevent the possible spread of HIV, pharmacists should maintain proper hygiene when contacting patients with the virus or dispensing medication to them. Using sterile procedures and providing education on safe disposal are also necessary. Therefore, possessing adequate knowledge of the disease, overcoming negative attitudes, and eliminating social discrimination against HIV-infected patients is crucial. Today’s pharmacy students are tomorrow’s professionals, and having positive attitudes toward HIV-infected patients can help in treating them fairly and professionally.

Regarding students’ attitudes toward HIV, current findings reveal that only 13.2% of students showed a positive attitude, while 61.5% had a neutral attitude. Additionally, 52.4% of students agreed that they were qualified to treat and care for patients with AIDS. Notably, 41% of pharmacy students expressed a lack of preference for treating HIV patients, attributing this to the significant stigma associated with individuals living with HIV and those at risk, including people who inject drugs and members of the LGBTQ+ community. The findings indicate an increased barrier for health workers in the region. Although the inconsistency of these findings among Chinese students is acknowledged, numerous other studies have demonstrated a considerable level of stigma toward people living with HIV and those at risk of HIV. A prior study indicated that 84.7% of Chinese college students exhibited a positive attitude toward HIV (18). Similarly, a recent study of pharmacy students revealed that 18.5% of them agreed they could provide HIV-positive patients with counseling and treatment (3). Therefore, possessing adequate knowledge of the disease, overcoming negative attitudes, and eliminating social discrimination against HIV-infected patients are crucial. Today’s pharmacy students are tomorrow’s professionals, and having positive attitudes toward HIV-infected patients can help in treating them fairly and professionally.

There are some limitations to the current investigation. First off, the fact that the results were based on a self-administered online survey, may have raised the likelihood of social desirability bias or recollection bias. Second, the study was limited to a single university, in the region of Saudi Arabia, and did not include the entire pharmacy’s student forces, which may restrain the generalizability of the findings globally. Despite these drawbacks, our study proposes placing a greater emphasis on educating health college students about HIV and dispelling myths about getting diseases when treating or dispensing medication to HIV-positive patients and providing them with the knowledge necessary to educate the general public about the disease. Therefore, education institutions should strengthen and promote the education of AIDS transmission and prevention.

## Conclusion

In conclusion, there was a dearth of knowledge of HIV/AIDS and neutral attitudes and perceptions of the disease (HIV) among pharmacy students. When compared to other age groups, students between the ages of 23 and 26 had a high degree of knowledge. Furthermore, compared to other students, intern students demonstrate a high level of knowledge about HIV/AIDS, and second-year students have a more positive perception of the risk of HIV/AIDS. These findings suggest that specific strategies, such as integrating education on this topic into the pharmacy curriculum and running awareness campaigns for undergraduate students, are necessary to minimize knowledge gaps regarding HIV/AIDS and combat negative attitudes and perceptions.

## Data Availability

The original contributions presented in the study are included in the article/supplementary material, further inquiries can be directed to the corresponding author.

## References

[1] World Health Organization. Global HIV, hepatitis and STIs programmes. HIV data Stat. Geneva: WHO (2020).

[2] World Health Organization. HIV and AIDS. International: World Health Organization. (2024). Available at: https://www.who.int/news-room/fact-sheets/detail/hiv-aids (Accessed November 1, 2024).

[3] Alzahrani F Almohammadi A Alhejaili M Almukhlifi S Alhudhayban A Khan A. An evaluation of pharmacy students’ knowledge, attitudes and risk perceptions about HIV/AIDS. Pharm Pract. (2024) 22:1–8. doi: 10.18549/PharmPract.2024.3.2998

[4] Qashqari FS Alsafi RT Kabrah SM RA AG Naeem SA Alsulami MS. Knowledge of HIV/AIDS transmission modes and attitudes toward HIV/AIDS infected people and the level of HIV/AIDS awareness among the general population in the kingdom of Saudi Arabia: a cross-sectional study. Front Public Health. (2022) 10:955458. doi: 10.3389/fpubh.2022.955458, PMID: 36238245 PMC9551601

[5] Business atlas. King dom of Saudi Arabia. (2024). Available at: https://atlas.monshaat.gov.sa/en/profile/country/saudi-arabia (Accessed November 1, 2024).

[6] Ahmed M: Higher education in KSA: changing demand in line with vision 2030. Saudi Arabia: Colliers (2022).

[7] Yang Y-m Shen Y-l Li S-y. Occurrence of sexual behavior among college students in mainland China: a meta-analysis. Chin J Public Health. (2018) 34:142–7. doi: 10.11847/zgggws1113830

[8] Al-Mozaini M Al-Rahabani T Dirar Q Alashgar T Rabaan AA Murad W. Human immunodeficiency virus in Saudi Arabia: current and future challenges. J Infect Public Health. (2023) 16:1500–9. doi: 10.1016/j.jiph.2023.06.012, PMID: 37353430

[9] Saudi Arabia-Prevalence Of HIV. Total (% Of Population Ages 15-49). Available at: https://tradingeconomics.com/saudi-arabia/prevalence-of-hiv-total-percent-of-population-ages-15-49-wb-data.html#:~:text=Ages%2015%2D49)-,Prevalence%20of%20HIV%2C%20total%20(%25%20of%20population%20ages%2015%2D49,compiled%20from%20officially%20recognized%20sources (Accessed November 1, 2024).

[10] Asrina A Ikhtiar M Idris FP Adam A Alim A. Community stigma and discrimination against the incidence of HIV and AIDS. J Med Life. (2023) 16:1327–34. doi: 10.25122/jml-2023-0171, PMID: 38107709 PMC10719780

[11] Terra M Okereke PU Wandera F Edith K Kitonga MS Ally AM. HIV related knowledge and practices among undergraduate students in Africa: a cross-sectional multinational study. J Med Surg Public Health. (2024) 3:100126. doi: 10.1016/j.glmedi.2024.100126

[12] Alawad M Alturki A Aldoghayyim A Alrobaee A Alsoghair M. Knowledge, attitudes, and beliefs about HIV/AIDS and people living with HIV among medical students at Qassim University in Saudi Arabia. Int J Health Sci. (2019) 13:22–30. PMID: 31501649 PMC6728128

[13] Black E Wilby K Perepelkin J. A survey of HIV knowledge and attitudes of pharmacy students in Canada and Qatar. Saudi J Health Sciences. (2013) 2:146–50. doi: 10.4103/2278-0521.127038

[14] Ahmed SI Hassali MA Aziz NA. An assessment of the knowledge, attitudes, and risk perceptions of pharmacy students regarding HIV/AIDS. Am J Pharm Educ. (2009) 73:15. doi: 10.5688/aj730115, PMID: 19513153 PMC2690879

[15] Bloom BS. Learning for mastery. Instruction and curriculum. Regional education Laboratory for the Carolinas and Virginia, topical papers and reprints, number 1. Evaluation. (1968) 1:n2

[16] Alali FM Tarakji B Alqahtani AS Alqhtani NR Nabhan AB Alenzi A. Assessment of knowledge and attitude of dental students towards HIV and its Oral manifestations in Saudi Arabia—a cross-sectional study In: Healthcare: MDPI (2022). 1379. doi: 10.3390/healthcare10081379 PMC9330909 35893201

[17] Zhang L Yu H Luo H Rong W Meng X Du X. HIV/AIDS-related knowledge and attitudes among Chinese college students and associated factors: a cross-sectional study. Front Public Health. (2022) 9:804626. doi: 10.3389/fpubh.2021.804626, PMID: 35096751 PMC8790097

[18] Ma Z Guo L Zhou M Zuo H. HIV/AIDS-related knowledge and attitudes towards HIV rapid testing among Chinese college students: findings from a cross-sectional survey. Prev Med Rep. (2023) 36:102409. doi: 10.1016/j.pmedr.2023.102409, PMID: 37719792 PMC10502351

